# Improving quality of care through improved audit and feedback

**DOI:** 10.1186/1748-5908-7-45

**Published:** 2012-05-18

**Authors:** Sylvia J Hysong, Cayla R Teal, Myrna J Khan, Paul Haidet

**Affiliations:** 1 Houston VA Health Services Research & Development Center of Excellence, Michael E. DeBakey VA Medical Center, Houston, TX, USA; 2 Department of Medicine – Health Services Research Section, Baylor College of Medicine, Houston, TX, USA; 3 Department of Medicine and Office of Undergraduate Medical Education, Baylor College of Medicine, Houston, TX, USA; 4 Department of Medicine, Penn State University College of Medicine, Hershey, PA, USA

## Abstract

**Background:**

The Department of Veterans Affairs (VA) has led the industry in measuring facility performance as a critical element in improving quality of care, investing substantial resources to develop and maintain valid and cost-effective measures. The External Peer Review Program (EPRP) of the VA is the official data source for monitoring facility performance, used to prioritize the quality areas needing most attention. Facility performance measurement has significantly improved preventive and chronic care, as well as overall quality; however, much variability still exists in levels of performance across measures and facilities. Audit and feedback (A&F), an important component of effective performance measurement, can help reduce this variability and improve overall performance. Previous research suggests that VA Medical Centers (VAMCs) with high EPRP performance scores tend to use EPRP data as a feedback source. However, the manner in which EPRP data are used as a feedback source by individual providers as well as service line, facility, and network leadership is not well understood. An in-depth understanding of mental models, strategies, and specific feedback process characteristics adopted by high-performing facilities is thus urgently needed.

This research compares how leaders of high, low, and moderately performing VAMCs use clinical performance data from the EPRP as a feedback tool to maintain and improve quality of care.

**Methods:**

We will conduct a qualitative, grounded theory analysis of up to 64 interviews using a novel method of sampling primary care, facility, and Veterans Integrated Service Network (VISN) leadership at high-, moderate-, and low-performing facilities. We will analyze interviews for evidence of cross-facility differences in perceptions of performance data usefulness and strategies for disseminating performance data evaluating performance, with particular attention to timeliness, individualization, and punitiveness of feedback delivery.

**Discussion:**

Most research examining feedback to improve provider and facility performance lacks a detailed understanding of the elements of effective feedback. This research will highlight the elements most commonly used at high-performing facilities and identify additional features of their successful feedback strategies not previously identified. Armed with this information, practices can implement more effective A&F interventions to improve quality of care.

## Background

The Institute of Medicine (IOM) strongly advocates the use of performance measures as a critical step toward improving quality of care [1-3]. Clinical performance measures systems such as RAND Corporation’s Quality Tools [4] and the External Peer Review Program (EPRP) of the Department of Veterans Affairs (VA), which monitors highly prevalent, high-impact clinical conditions and preventive processes such as diabetes, hypertension, cancer screening, and tobacco cessation counseling, [5] have led to significant improvements in quality of care [6,7]. Despite these improvements, research shows significant variability across clinical quality measures over time in both the mean performance level in any one quarter and the degree of improvement over multiple quarters [8].

Audit and feedback (A&F), an important component of effective performance measurement and pay-for-performance programs according to the IOM, [3,8] has been found to be among the most effective behavioral interventions for improving care quality in numerous settings and can help reduce variability and improve overall quality of care[9-11]. Recently, A&F has gained renewed attention due to its essential role in effectiveness of and attitudes toward emerging physician-based performance measurement and pay-for-performance initiatives [12,13]. A&F has also been suggested as an important component in continuing education, as research has shown physicians have limited ability to accurately assess their continuing education needs [14]. Consequently, healthcare organizations, providers, and patients alike thus stand to gain significantly from a well-designed and well-implemented A&F intervention.

Recent research has made some important insights in how feedback works in the healthcare setting in general [10,15] and has also noted that healthcare facilities that more successfully implement evidence-based guidelines tend to use clinical performance data as an essential source of feedback [16,17]. However, the extent to and manner in which clinical performance data are used as a feedback source by individual providers, service line chiefs, or facility leaders is not well understood*.* Through the research outlined in this protocol, we aim to gain an in-depth understanding of perceptions, strategies, and specific feedback process characteristics adopted by high-, moderate-, and low-performing primary care facilities.

### Conceptual model: an actionable model of feedback

A&F research in healthcare has been criticized as being atheoretical; [9,11] as Foy and colleagues note, we have “an inadequate understanding of the causal mechanisms by which [A&F] or its variants might exert their effects” [18]. Several theoretical perspectives, including Diffusion of Innovations theory, motivational theories, and Feedback Intervention Theory (FIT), could shed light on how to maximize feedback effectiveness. Rogers’ Diffusion of Innovations model [19] is commonly applied to the evaluation of quality-improvement interventions in healthcare such as A&F; however, as adoption is not the subject of the current proposal, Rogers’ model is inappropriate for our needs.

Another theoretical perspective worth considering is provider motivation. Both classic motivational theories, such as expectancy theory [20], and more recent work, such as goal-setting theory [21], suggest feedback is an important moderator of individual motivation. However, empirical research suggests that not all feedback is created equal—several characteristics of both the individual and the feedback itself have been long documented to affect subsequent performance;[22-25] yet both of these motivational theories treat feedback as either present or absent.

**Figure 1:**
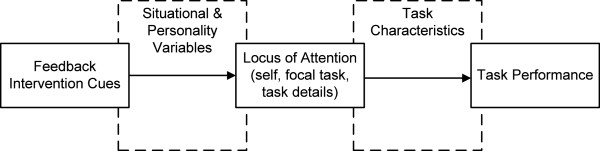
Schematic of Feedback Intervention Theory tenets.

Kluger and DeNisi’s FIT,[26] a well-documented framework from industrial/organizational psychology theorizing about the intervention itself in addition to the recipients of the intervention, is thus the best theoretical choice for guiding our research design and accomplishing our scientific objectives. In brief, FIT posits that feedback interventions work by providing new information that redirects recipients’ locus of attention either away from the task (*e.g.*, toward ourselves or toward irrelevant tasks) or toward the details of the task. Information that redirects attention toward the details of the task tends to strengthen the feedback’s effect on task performance; information that shifts attention away from the task tends to weaken this effect [26]. Consequently, Kluger and DeNisi proposed that three factors determine how effectively this attentional shift occurs: characteristics of the feedback itself (or “feedback intervention cues” in FIT parlance), task characteristics, and situational variables. Figure 1 graphically depicts the basic tenets of FIT. 

**Figure 2:**
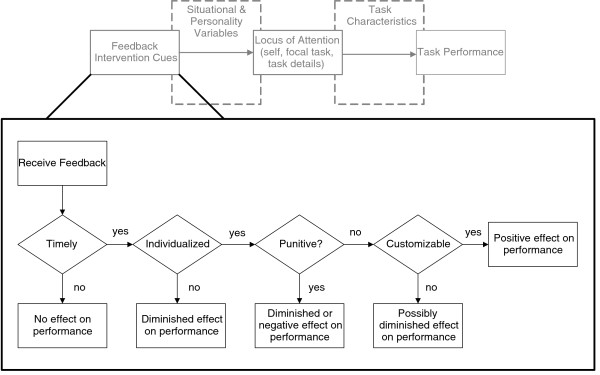
**Hysong*et al.*’s (2006) model of actionable feedback in the context of Feedback Intervention Theory.**

Of the three factors proposed by FIT, feedback intervention cues are the easiest to change; simple changes to the format, frequency, and message of the feedback delivered could result in notable differences in performance across a wide variety of tasks and providers. However, Kluger and DeNisi found as many as seven different cues significantly impacting performance, with little guidance as to how to prioritize these cues. Based on a grounded theory–based exploration of barriers and facilitators to clinical practice guideline implementation, Hysong and colleagues propose a model of actionable feedback that explores the relationships amongst a subset of feedback cues in more detail (Figure 2) [15]. According to the model, three cues are necessary prerequisites to effective feedback: timeliness, individualization, and nonpunitiveness. These cues are sequentially arranged in the model, with those cues providing the most barriers to feedback effectiveness (*i.e.*, those which, if absent, would pose the greatest barriers to feedback effectiveness) listed first. For example, the most customized, individualized, nonpunitive feedback intervention is useless if the provider does not receive it in time to make a change. Each cue provides increased meaning to the feedback (Is it on time? Is it about me? Is it constructive?), thus making the feedback increasingly actionable. A fourth cue, customizability, while not a prerequisite, is considered a significant enhancer. That is, by affording the user the ability to process the feedback in a way most meaningful to him/her, it enhances the actionability of the feedback. 

Using the Hysong et al. model as a guiding framework, this research (a) evaluates the perceived utility of clinical performance measures as a viable tool for delivering clinical performance feedback to providers; (b) examines differences in how leaders of high-, moderate-, and low-performing facilities use EPRP for such a purpose; and (c) seizes the opportunity to test the fit of the Hysong et al. model in a larger, independent sample specifically designed to investigate feedback strategies within the VA. Through this research, we aim to answer the following research questions:

 1. How do leaders of high-performing facilities perceive clinical performance measures data as a source of feedback, and how do their perceptions differ from leaders of low and moderately performing facilities?

 2. What strategies do leaders of high-performing facilities employ to collect and disseminate performance data, and how do these differ from those of low and moderately performing facilities?

 3. Do high-performing facilities share their data in a more timely, individualized, and nonpunitive fashion than do low and moderately performing facilities?

## Methods/Design

### Design

**Figure 3:**
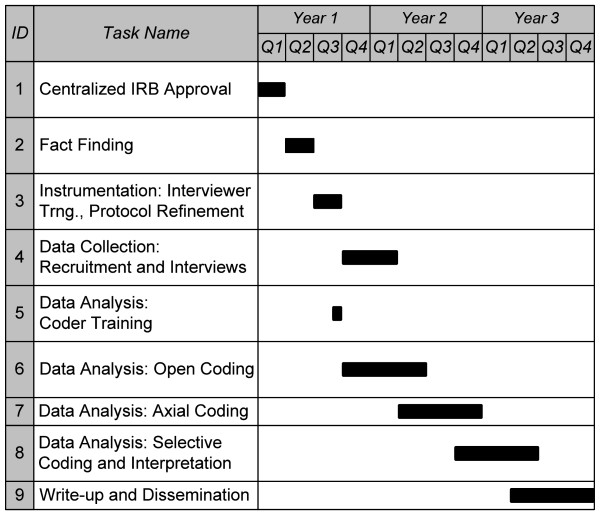
Projected study timeline.

The proposed research consists of qualitative, primary analysis of telephone interviews with regional and facility leadership and primary care personnel at 16 VA Medical Centers (VAMCs), employing a cross-sectional design with purposive sampling guided by preliminary analyses of clinical performance data. The study is projected to take three years to complete. Figure 3 presents a timeline of the various stages of the process, each of which is described below.

### Sample

#### Site selection

##### Site-sampling strategy

Recent research suggests that the greatest practice variation occurs at the facility level [27,28]. Therefore, sites were selected using a purposive stratified approach based on their performance on a profile of 15 outpatient EPRP measures (see Data Source for Site Selection subsection, below)^a^.

A common strategy for this kind of research is to select high- and low-performing sites, as it maximizes the researcher’s ability to observe differences amongst the strata in the construct of interest. However, this strategy assumes some form of linear relationship—that is, that those unobserved sites falling between the high- and low-performing strata will exhibit the construct of interest more strongly than those in the low strata but less strongly than those in the high strata. However, as discussed in more detail below, there are multiple ways of achieving moderate performance, and we cannot assume that all moderately performing facilities will exhibit the constructs of interest in the same way. Consequently, we will identify four high-performing, four low-performing, and eight moderately performing (four consistently average and four highly variable) facilities.

##### Data source for site selection

Performance data for site-selection purposes will be extracted from the EPRP data warehouse. The EPRP is the official data source for the VA’s clinical performance management system and provides the indicators that Veterans Integrated Service Network (VISN) directors use to gauge performance and make administrative decisions about facilities in their networks. The EPRP is a nationally abstracted database containing performance data for all VA medical facilities on over 90 indicators covering access, quality of care, cost effectiveness, and patient satisfaction domains [29]. Data for calculating these performance indicators are abstracted directly from patient charts by the West Virginia Medical Institute using explicit abstraction rules and a rigorous, well-documented oversight and inspection process to ensure data accuracy and quality [29,30]. Independent analyses of EPRP data indicate high interrater reliability (kappa = .90) [31]. The objective of this database (dating from FY2000 and updated quarterly) is to assess the quality of care delivered in the VA and to allow comparisons of individual VAMCs and VISNs to the VA system as a whole, to private and public sector hospitals, and to the optimum standard of care. Data elements in the quality-of-care domain include indicators for cancer, diabetes, cardiovascular conditions, infectious diseases, and mental health, among others. Table 1 presents the specific EPRP measures used to calculate the facility performance composites. 

**Table 1:** List of EPRP measures used to create facility performance profiles

**EPRP mnemonic**	**Short description**
c7n	DM - Outpatient - Foot sensory exam using monofilament
Dmg23	DM - Outpatient - HbA1 > 9 or not done (poor control) in past year
Dmg28	DM - Outpatient - BP > =160/100 or not done
Dmg31h	DM - Outpatient - Retinal exam, timely by disease (HEDIS)
Dmg7n	DM - Outpatient - LDL-C < 120
htn10	HTN - Outpatient - Dx HTN & BP > = 160/100 or not recorded
htn9	HTN - Outpatient - Dx HTN & BP < = 140/90
p1	Immunizations - Pneumococcal Outpatient – Nexus
p22	Immunizations - Outpatient - Influenza ages 50–64 - Nexus clinics
p3h	CA - Women aged 50–69 screened for breast cancer (HEDIS)
p4h	CA - Women aged 21–64 screened for cervical cancer in the past three years (HEDIS)
p6h	CA - Patients receiving appropriate colorectal cancer screening (HEDIS)
smg2n	Tobacco - Outpatient - Used in past 12 months - Nexus - Non-MH
smg6	Tobacco - Outpatient - Intervention - Annual - Non-MH with referral and counselling
smg7	Tobacco - Outpatient - Meds offered - Nexus - Non-MH

Note. The EPRP mnemonic is an identifier in the database for a given measure calculated in a given way. OQP may, over time, change the criteria for a given measure. A measure attempting to capture a similar outcome but with a different calculation method would be assigned a different mnemonic number (for example, dmg28 in the above table, currently described as BP greater than or equal to 160/100, was listed as BP greater than 160/100 in earlier years and would have received a different mnemonic identifier). For purposes of this study, only measures with identical mnemonic during *all* quarters of the calculation period were used.+.

*EPRP* External Peer Review Program; *DM* Diabetes Mellitus; *BP* blood pressure; *HEDIS* Healthcare Effectiveness Data and Information Set; *HTN* Hypertension; *CA* Cancer; *MH* mental health; *OQP* Office of Quality and Performance.

##### Measuring facility performance for site classification

Once a sampling strategy has been selected, the next challenge is operationalizing and measuring the variable or feature by which the sites are to be selected. In the case of facility performance, a common strategy is to create composite scores of performance based on a set of individual measures (in this case, the EPRP measures). However, examining moderate performance using composites poses a particular challenge, as multiple profiles of moderate performance exist. Facilities can exhibit moderate scores across most or all measures of performance (consistently moderate); alternatively, facilities can average out to moderate performance by performing highly on some measures and poorly on others (highly variable). A composite measure of performance would categorize both of these cases equally as moderate performers, yet they represent considerably different facilities. A more novel and effective approach, and the one used herein, is to select facilities based on their pattern of performance on a selection of individual measures rather than based on a composite, as described below.

For this research, we will measure facility performance using a profile of 15 outpatient EPRP measures (listed in Table 1) over the two-year period of FY2007–2008 to classify facilities into high, moderate, and low performers and, thus, identify the sites to be recruited. Though variation exists across facilities in performance, the range of scores is nonetheless relatively restricted, as initiatives such as implementing target scores and floors on performance measures have improved facility performance over time. However, initial examination of the data revealed that no facilities were either consistently stellar or consistently abysmal across all measures. Thus, our selection criteria are based on more realistic, yet sufficiently stringent, expectations given the data, to balance the need to minimize overlap in performance between high-, moderate-, and low-performing facilities with the performance realities of the data set.

*High-performing facilities* will be defined as those whose two-year scores for each EPRP measure are at the 84th percentile or higher. We will sort facilities by the number of EPRP measures meeting this criterion and target the four facilities with the greatest number of measures meeting this criterion.

*Low-performing facilities* will be defined as those whose two-year scores for each EPRP measure are at the 16th percentile or lower. We will sort facilities by the number of EPRP measures meeting this criterion and target the four facilities with the greatest number of measures meeting this criterion.

Unlike the profile criteria for high- and low-performing facilities, profiles for the moderate-performing facilities are much more difficult to devise on a pattern of individual measures, requiring visual pattern matching that could prove unsystematic. However, measures of dispersion, such as the standard deviation, provide a suitable gauge for distinguishing between consistently moderate and highly variable performers and form the basis of our selection criteria for moderate performers. First, we will average all the EPRP measure scores for each facility, create an average facility performance score (AFPS), and select the subset of facilities whose AFPS falls between the 35th and 65th percentile. This initial step is necessary to ensure comparability of sites—in other words, we want to select sites that, overall, perform “moderately”; from those sites, we can then distinguish consistently moderate from highly variable facilities using our measure of dispersion. To accomplish this, we will create a performance variability score (PVS) for each facility, composed of the standard deviation of their individual performance measure scores.

*Consistently moderate performers* will be defined as the four facilities with the lowest PVS from the subset of facilities with an AFPS between the 35th and 65th percentiles. *Highly variable performers* will be defined as the four facilities with the highest PVS from the subset of facilities with an AFPS between the 35th and 65th percentiles.

Table 2 lists basic descriptive characteristics for all study sites sampled. Specific site names have been omitted to maintain blinding and confidentiality; the list includes facilities from the east coast, midwest, and southwest.

**Table 2:** Descriptive statistics for study sites

**Facility type**	**Size (number of unique patients)**	**Residents per 10 k patients**^**a**^	**Primary care presence**^**b**^	**Number of primary care personnel**
HIGH	27,222	0.00	0.12	35
	49,813	31.42	0.26	83
	44,114	0.23	0.37	56
	27,851	8.62	0.14	62
MODERATE: consistently average	63,313	10.63	0.66	94
	75,609	18.83	0.08	115
	62,017	21.58	0.01	125
	51,645	30.70	0.33	54
MODERATE:highly variable	63,555	14.81	0.21	30
	27,222	0.00	0.12	143
	72,739	35.06	0.45	27
	14,149	0.00	0.28	10
LOW	58,630	24.94	0.16	116
	24,795	0.00	18.02	23
	19,609	0.00	0.10	46
	44,391	27.51	0.12	88

^a^A measure of the strength of the facility’s academic orientation. Greater numbers mean a stronger academic orientation; 0 means no academic affiliation. ^b^Operationalized as the percent of outpatient clinic stops provided at community-based outpatient clinics (a low percentage indicates the majority of outpatient/primary care occurs at the main hospital, rather than at satellite clinics).

Source: Daw et al., 2009[32].

### Participants

We will interview four informants at each facility, selected on the basis of availability, from the following groups: the facility director, the associate chief of staff (ACOS) for primary care, one full-time primary care physician and/or physician extender, and one full-time primary care nurse, for *up to* 64 interviews (or until saturation across key informant type is reached). New or part-time employees would not have sufficient exposure to the EPRP to make informed appraisals of its utility; therefore, we will only target full-time primary care providers with at least three years in their current position. We will query the Personnel Accounting Integrated Database (PAID) to identify eligible participants who meet these criteria. Primary care participants will then be randomly selected from the resulting list and invited to participate via email. We will confirm the eligibility of potential participants upon invitation to participate. Every effort will be made to ensure a balanced representation of informants across facilities. If a participant declines to participate, we will ask them to refer us to another suitable candidate.

### Procedures

#### Research team and participant blinding

The proposed research will use a double-blind design. The team statistician will be the only member of the research team with knowledge of which facilities are high, moderate, or low performers. All other team members, including co-investigators and research assistants, will be blinded to minimize bias during the interviewing and data analysis processes. Similarly, participants will also be blind to their facility’s performance category so as to minimize respondent bias.

#### Interviewer training

Interviewers will receive training consisting of three components, consistent with the Information, Demonstration, Practice (IDP) framework of training delivery: [33]

 1. a didactic training session (*information*) by Dr. Haidet,

 2. observation of interviews conducted by Drs. Haidet and Hysong (*demonstration*), and

 3. two mock interviews (*practice*).

#### Preparatory facility fact finding

The research team will conduct telephone fact-finding interviews with key contacts at each study facility to gather factual information about the facility’s EPRP dissemination process, such as the type of performance measurement data used by the facility or whether the facility uses a locally generated dashboard. This information will (a) provide greater contextual understanding of existing facility processes related to EPRP, (b) help refine the telephone interview guide, and (c) help the research team identify the best strategy for study interviews. Examples of key contacts are the Facility Quality Manager and ACOS and/or their designee(s). We will use a snowball contact-and-referral process until all the requisite factual information is obtained for each facility. For example, if our first contact at the facility is unable to provide the survey questions, the research team will request the name of another individual at the facility who is more likely to have the answers, and so on.

Each telephone conversation will take approximately 30 minutes and will include topics about the dissemination processes and reporting of EPRP data at the facility. Interviewers will follow a standardized question guide about the dissemination process of EPRP data at their facility.

#### Participant recruitment

Prospective participants will receive an email inviting them to participate in the study and requesting a preferred contact email, phone number, and a time where a representative of the study team can review the study information form with them and obtain informed consent. Prospective participants who have not responded to the invitation within two weeks will receive a follow-up telephone call inviting them to participate; the aforementioned contact and scheduling information will be collected from these participants at that time. Research team members will email a copy of the study information form in advance and will call the participants at their requested appointment time to review the consent form and procedures with the participant, answer any questions, and schedule the interview. Should a prospective participant decline the invitation and not provide a recommendation for a substitute participant (see Participants section, above), the next prospective participant on the list of candidates will be invited.

As much as possible given participant schedules, interviews will be scheduled following a maximum variation strategy at the facility level. The statistician will provide specific site names to the study team members for recruitment purposes, ensuring that all four arms are represented in the resulting list of sites. Research team members will then schedule and conduct interviews until all interviews are completed or saturation of information is reached within study arms, whichever comes first.

#### Telephone interviews

Participants will be interviewed individually for one hour by a research assistant at a mutually agreed upon time. All interviews will be audio-recorded with the participant’s consent. The interview consists of two initial “picture questions,” which ask the respondent to provide an example of a feedback strategy that resulted in practice change and an example of a feedback strategy that did not result in practice change. The answers to these two questions will guide the rest of the interview. Based on the participant’s initial answers, interviewers will ask follow-up questions that will tap the constructs of interest in the study. Additional file 1 presents a preliminary interview protocol listing the constructs of interest and their corresponding proposed questions for each type of key informant. The interviewers need not ask the questions in the order listed nor use all of the probing questions; however, they are required to cover all of the constructs of interest. Participants will answer questions about (a) the types of EPRP information they receive, (b) the types of quality/clinical performance information they actively seek out, (c) opinions and attitudes about the utility of EPRP data (with specific emphasis on the role of targets), (d) how they use the information they receive and/or seek out, and (e) any additional sources of information or strategies they might use to improve the facility’s performance. Interview recordings will be sent for transcription the day after the interview; we will begin analyzing transcripts per our data analysis strategy when they are received.

To minimize participant burden, interviews will be scheduled and analyzed concurrently until saturation of information is reached. That is, up to 64 interviews may be conducted, but fewer may be conducted if no new information is encountered. In order to check for thematic saturation, we will code and analyze interview transcripts as we receive them, rather than wait until all interviews are conducted. As new codes indicate new concepts of importance, a lack of new codes is indicative that no new information is being generated by additional interviews and that the data are sufficiently saturated. We will end the interview process when a new interview adds less than 5% of the total number of existing codes in new codes; for example, if 100 codes have been generated after 25 interviews, and the 26th interview only adds four new codes, we will consider the data to have reached saturation and end the interview process.

#### Data analysis

Interview recordings will be transcribed and analyzed using techniques adapted from grounded theory [34] and content analysis [35] using Atlas.ti (ATLAS.ti Scientific Software Development GmBH, Berlin, Germany)*,*[36] a qualitative analysis software program. Consistent with grounded-theory techniques, the analysis will consist of three phases: open, axial, and (if appropriate) selective coding.

#### Coder training

Before the coding process begins, the principal investigator will conduct a training session (consistent with the IDP framework) with the coders and co-investigators to familiarize them with the Atlas.ti software and the initial coding taxonomy. The session will consist of two modules:

 1. A didactic module, where the trainees will receive detailed information about the specific *a priori* codes to be searched for in the texts (*e.g.*, definitions, examples, negative cases), guidelines for identifying new themes and codes, and a demonstration of the Atlas.ti software features and its project-specific use.

 2. A practice module, where coder teams will use the mock interviews from their interviewer training practice module to practice coding and calibrate the coders to the taxonomy of utility perceptions, strategies, and data-sharing practices.

In addition, coders will independently code two live transcripts and then convene to discuss their coding decisions, in order to further calibrate the coders on live data.

#### Open coding

Open coding is concerned with identifying, naming, categorizing, and describing phenomena found in the interview transcripts. The same research assistants who conducted the interviews will conduct the open-coding phase of analysis. Each research assistant will independently code all interview transcripts; each will serve as primary coder for the interviews they conducted and as secondary (*i.e.*, corroborative) coder for interviews they did not conduct. Secondary coding assignments will be distributed such as to maximize the number of different coders reviewing the transcripts of any given site.

Coders will receive an *a priori* list of codes and code definitions, designed to capture the relevant constructs of interest for the study. These include (but are not limited to) feedback cues regarding content and format as specified in Kluger and DeNisi, 1996 (*e.g.*, correct solution information, frequency); feedback characteristics as specified in Hysong et al., 2006 (*i.e.*, timeliness, punitiveness, individualization, customizability); attitudes and mental models about EPRP (*e.g.*, positive/negative, concerns of trust or credibility of feedback); and feedback sources. Based on this list, coders will select relevant passages indicative of a given phenomenon (*e.g.*, timely sharing of performance information) and assign it a label descriptive of the phenomenon in question (*e.g.*, “timeliness”). Coders will specifically review the transcripts for instances of the constructs involved in the research questions (perceptions of EPRP utility as a feedback source; local data collection, dissemination and evaluation strategies; timeliness, individualization, and nonpunitiveness of data sharing) and capture them as they emerge from the data.

Coders will also have the opportunity to add new codes to the existing list. Proposed codes will be vetted by the research team according to two criteria: (1) whether the codes can be clearly and crisply defined and (2) the extent to which the new codes contribute to the analysis. Codes deemed to be suitable will then be added to the list; previous transcripts will be coded with the new codes as needed.

#### Ensuring coding quality

Once a transcript is coded by the primary coder, a secondary coder will independently review the primary coder’s assignments and agree or disagree with each code attached to a quote. To minimize potential bias, pairs of coders will rotate—that is, a given primary coder will not have all his/her transcripts reviewed by the same secondary coder; complete rotation of primary and secondary coder pairs can be accomplished in 24 (4!) interviews. Counts of “agree” and “disagree” code assignments will be tallied to help estimate the extent of inter-rater agreement and identify codes or themes that require crisper definition if necessary. After the secondary coder’s review, any disagreements will be resolved by consensus between the primary and secondary coder. Any disagreements that the coder pairs could not resolve will be presented to the entire team for resolution.

Other data quality checks will be performed, including tests of quotation length over time (significant increases in quotation lengths in later portions of a transcript or in later transcripts could be indicative of coder fatigue) and between raters (to check for biases in coding styles).

#### Axial coding

In this phase, the categories generated during the open coding phase are organized into coherent themes and networks of relationships using the “constant comparative approach”;[34] the investigator team will review the generated codes, noting their frequency and searching for relationships among the codes as they emerge.

Research question 1 asks whether leaders of high-performing facilities have different perceptions about the utility of facility performance data than do leaders of low- or moderate-performing facilities. To explore this research question, the investigator team will review the codes generated in relation to this question and compare the universe of perceptions in high-, low-, and moderate-performing facilities. These will be organized thematically, with separate taxonomies and relational networks (*i.e.*, visual maps of how the codes relate to one another) developed for each facility type*.* To the extent the corpus of codes is different across facility types (*e.g.*, a greater number of nonoverlapping codes than codes in common), or that similar codes exist in greater or less frequency in high- versus low-performing facilities, this will be taken as evidence that high-performing facilities perceive the utility of EPRP data differently than do low-performing facilities.

Research question 2 asks whether leaders of high-performing facilities employ different strategies than leaders of low- or moderately performing facilities to collect and disseminate local performance data and to evaluate providers. This research question will be explored using a parallel approach to that of research question 1*.* The investigator team will review the codes generated in relation to the questions in the interview protocol about feedback strategies and compare the universe of strategies in high-, moderate-, and low-performing facilities. These will be organized thematically, with separate taxonomies and relational networks developed for each facility type. To the extent that there are a greater number of discrepant themes across subgroups than there are themes in common, this will be taken as evidence that (a) different facility types employ different strategies than low-performing facilities to collect and disseminate local performance data and to evaluate providers and (b) the core category emergent from the selective coding process differs by subgroup.

#### Selective coding

This stage of the analysis involves selecting one of the categories developed during axial coding as the core category around which the theory best fitting the data is to be built. In other words, there should be a central theme emergent from the data best *explaining* the relationships among the other categories (codes). Hysong et al.’s model of actionable feedback [15] was developed in this way, with the concept of customizability as the core category. This phase of the analysis will be most useful in exploring research question 3.

Research question 3 asks whether high-performing facilities share their data in more timely, individualized, and nonpunitive ways than do low- or moderate-performing facilities. To explore this research question, the investigator team will review codes generated in relation to the questions in the interview protocol about timeliness, individualization, and actionability of feedback, which captured instances where facilities delivered performance data to its providers in a timely (*e.g.*, at least monthly), individualized (provider-specific, rather than aggregated by clinic or facility), and nonpunitive fashion (*e.g.*, mention of educational, developmental approaches to feeding back the data to providers). These will be organized thematically, with separate taxonomies and relational networks developed for each facility type (axial coding, as described above). From this relational network, a core category will emerge around which a model of feedback can be organized; we will compare each subgroup’s emergent model to Hysong et al.’s model of actionable feedback [15]. We expect that the feedback model for the high-performing facilities will more closely resemble the Hysong et al. model than the lower-performing facilities.

#### Maximizing confirmability and trustworthiness

Several techniques will be employed to minimize potential biases resulting from the differences in experiences of the interviewers and coders*.* Interviewers and coders will be trained using a standardized training protocol (see interviewer and coder training sections described earlier); as part of the training, interviewer assumptions and expectations will be documented prior to conducting any interviews. Assumptions and impressions generated during interview coding will be documented simultaneously with the originally planned coding as the interviews are coded. These assumptions and impressions will be constantly referenced and compared with planned codes during the coding process to check for bias. Lastly, negative case analysis will be conducted to check for evidence contradictory to the hypotheses. Together, these strategies will help maximize the analyses’ confirmability and trustworthiness.

#### Timeline

This research is projected to last three years.

## Discussion

### Contributions to science and practice

Most research examining feedback to improve provider and facility performance lacks a detailed understanding of the elements of effective feedback [9]. Initial progress in this area has already been made with a systematic review of feedback characteristics [10]. The proposed research goes further by (1) examining an important*,* yet relatively unexplored, element of feedback interventions, the mindset and environment of the feedback giver; (2) explaining some of the mixed results observed in the A&F literature; and (3) more importantly, providing an opportunity to examine all three components of FIT in a single study and to independently validate the propositions put forth by Hysong and colleagues [15] in their model of actionable feedback.

The more robust theoretical model resulting from the proposed research will lead to the design of better A&F interventions. We will search high-performing facilities for instances of feedback characteristics already shown to be beneficial (*e.g.*, frequency and timeliness; written, individualized feedback; suggestions for improvement) to explore current manifestations in live clinic settings. Additionally, we will search for additional features of their successful feedback strategies not previously identified. Armed with this information, practice will benefit from this research by gaining the tools needed to design and implement more effective A&F interventions that target the information needs of providers, thus improving quality of care.

### Limitations

The greatest limitation in the proposed research lies in the number of interviewees at each facility—at each facility we are only interviewing one person of each role type. In the case of the ACOS for primary care and the facility directors, only one such person exists for each role. However, adding more primary care personnel to the numbers currently planned would make the research cost- and schedule-prohibitive. Nonetheless, each informant will provide a needed and different perspective to the question of EPRP data use.

## Endnote

^a^Sites were selected as an activity preparatory to research, before the start of the study, and are therefore not itemized in the study timeline.

## Competing interests

The authors declare that they have no competing interests.

## Authors’ contributions

SJH is the principal investigator for the project, was responsible for the study design, and had principal writing responsibility for this manuscript. CRT is a co-investigator on the project; she made material contributions to the study design and made material edits to this manuscript. MJK is the statistician for the project; she was responsible for executing the site sampling strategy, is the keeper of the identity of the study sites, and made material edits to this manuscript. PH is the qualitative methodologist and clinician for the project. He was responsible for the finalized design of the interview guide, conducting interviewer and coder training, made material contributions to the design of the study, and made material edits to this manuscript. All authors read and approved the final manuscript.

## Supplementary Material

Additional file 1 Appendix: Interview guides. Click here for file
