# Supplementary material for: Improving quality of care through improved audit and feedback
Source: Implement Sci. 2012 May 18;7:45. doi: 10.1186/1748-5908-7-45 (PMC3462705; doi:10.1186/1748-5908-7-45)
Supplement: Additional file 1 — Appendix: Interview guides. [file 1748-5908-7-45-S1.doc]

# Appendix

# Interview Guide – Clinician*

**Italicized text* – present in nurse guide but not physician guide

*All other text – present in both physician guide and nurse guide

#### Intro / Rapport Building

| **Concepts Tapped** | **Question** | Probes |
| --- | --- | --- |
| **Warmup/rapport building** | 1. Tell me about your role at the VA. |  |
| **How they personally monitor their own performance -- precursor** | 1. In your efforts to provide the highest quality care that you can, how do you go about assessing the quality of care that you currently provide? | Directions you might potentially go here:   - What do you find helpful /useful about <whatever strategy they just said> - *What, if any, tools do you use to reflect on how well you and/or your team have cared for your patients? How are these the same/different from the tools used by your facility?* - *In terms of the clinical care you provide, what criteria do you use to assess the quality of care that you deliver?*   In response to “I know I’m doing well when my patients are happy and healthy,” ask as follow-up   - How do you go about finding that out? Or What is it that lets you know they are happy/healthy? |
| ***Floater: Ask at some point during interview.*** | *What role do you play in improving clinical performance at your facility/clinic?* |  |

#### Exploration Area #1: Perceptions of EPRP and Clinical Performance Measurement

| **Concepts Tapped** | **Question** | Probes |
| --- | --- | --- |
|  | 1. How is clinical performance measured in your facility? | In response to unqualified mention of performance measures:   - Which performance measures do you mean? - *How is clinical performance measured for nurses?* - *How is it communicated to nurses? (meetings, individually, in mailboxes, etc)*   In response to communication mode:   - How is the performance information discussed? |
| **EPRP Mental models**  **(External Peer Review Program)** | 1. When you hear ‘EPRP’, what comes to mind? | - Tell us your understanding of the EPRP process |
| **EPRP as feedback** | 1. How does EPRP fit in to the measurement of clinical performance at your facility? | - *As a nurse, what is your role regarding the performance measures in EPRP?* - *Tell me about a time when you had to gather information for EPRP/performance measures.* - *How does EPRP relate to you and your team?* - How does the EPRP feedback you receive help your thinking and/or help change your practice?   If perceived as useful:   - How does the EPRP data you receive help you with your clinical performance?   If perceived as administrative burden:   - In what way? |

#### Exploration Area #2: Feedback Strategies

| **Concepts Tapped** | **Question** | Probes |
| --- | --- | --- |
| **Feedback characteristics** | 1. Tell us about how you receive feedback about clinical performance at your facility. | - Does someone on the outside (completely external) give feedback? - Who conducts your annual performance review? What elements of your clinical performance are evaluated? By what means is your clinical performance assessed? - *Where do you get information about your clinical performance? What are the sources of that information?* - *Feel free to probe about giving/receiving formal/informal feedback.*   To confirm EPRP as only feedback source:   - In what ways other than through EPRP do you receive feedback about your clinical performance? |
|  | 1. Tell me about the last time you received feedback about clinical performance. | - If their answer did not include EPRP, then ask: Tell me about the last time you received EPRP data   If this isn’t going anywhere, it might help to focus it a little more:   - Tell me about the last time you received feedback about your clinical performance that compelled you to change your practice. S-B-O[[1]](#footnote-2): What was the situation? What feedback did you receive? What did you change? - Give me an example of feedback you received that did nothing to compel you to change your practice. (situation-behavior-outcome) |
| **Feedback acceptance** | 1. What do you personally do with the feedback when you get it? | - Do you (does your clinic, facility, etc.) develop an action plan for the feedback? - *How involved are you in deciding what should be done, if anything, in response to new clinical performance data?* - *What do you think people ought to do with the feedback that they get re: clinical performance?* - *In response to what types of feedback are you likely to initiate a change?* |
|  | 1. What are the consequences of feedback at your facility? (i.e., what happens if you are given feedback about your performance and you choose to ignore it) | - What avenues are there if you don’t agree with a piece of feedback you receive? (e.g., appeal, grievance, is it all just handled informally) |
| **Feedback seeking** | 1. What could your facility be doing that they’re not doing now to help you track your individual clinical performance? | - How would those things help you with your clinical performance? - *If you were to get feedback that would be most helpful in improving the quality of your performance what would that feedback be about? What content areas would you want feedback about that would most improve your performance as a nurse?* |
|  | 1. Is there anything else about feedback that we have not discussed that you would like to share? |  |

# Interview Guide – Leader*

**Italicized text* – to be used with ACOS of primary care but not facility director

* All other text – to be used with both ACOS of primary care and facility director

#### Intro / Rapport Building

| **Concepts Tapped** | **Question** | **Probes** |
| --- | --- | --- |
| **Warmup/rapport building** | 1. Tell me about your role at the VA. |  |
| **How they personally monitor their own performance -- precursor** | 1. In your efforts to ensure delivery of the highest quality primary care to veterans, how do you go about assessing the quality of care that your Facility currently provides? | What do you find valuable about <whatever strategy they just said>?  How do you set (or learn about) organizational priorities for your facility regarding clinical performance?  In response to “I know I’m doing well when my patients are happy and healthy,” ask as follow-up   - How do you go about finding that out? Or What is it that lets you know they are happy/healthy? |

#### Exploration Area #1: Perceptions of EPRP and Clinical Performance Measurement

| **Concepts Tapped** | **Question** | **Probes** |
| --- | --- | --- |
|  | 1. How is clinical performance measured in your facility? | What tools are most valuable for gauging (or improving) clinical performance? Why? |
| **EPRP Mental models** | 1. When you hear ‘EPRP’, what comes to mind? | You can preface by saying “People from different facilities may think differently about EPRP; when you hear those letters, what comes to your mind?  We know there is a “textbook” definition, we’re interested in your concept of EPRP.  What does EPRP mean for you?  Give me your understanding of what EPRP is all about.  Tell us your understanding of EPRP   - measures - data collection - report delivery - and how the results are used |
| **EPRP as feedback** | 1. How does EPRP fit in to the measurement of clinical performance at your facility? | In what ways has EPRP been useful?  In what ways has EPRP not been useful?  If EPRP is one of many sets of critical measures they are responsible for, ask about their, their Facility and/or VISN’s current and past priorities. To what extent are clinicians exposed to EPRP data? To what extent are they exposed to other data related to EPRP measures?  What mechanisms are in place for providers to learn about EPRP measures/data? What education is provided them about EPRP measures/data?  How is the process of EPRP data collection and use explained to clinical staff? |

#### Exploration Area #2: Feedback Strategies

| **Concepts Tapped** | **Question** | **Probes** |
| --- | --- | --- |
| **Feedback characteristics** | 1. Tell us about how you give *(and receive)* feedback about clinical performance at your facility. | How involved are you in delivering clinical performance feedback to others in the organization? If little to no involvement, to whom does this responsibility get handed off? How are these people held accountable?  By what means is feedback given? What led you to adopt that approach to feedback?  What data inform the feedback that is given?  To confirm EPRP as only feedback source:  In what ways other than through EPRP do you provide feedback about clinical performance? |
|  | 1. Tell me about the last time you gave feedback about clinical performance. | If their answer does not include distributing EPRP, then ask: Tell me about the last time you distributed EPRP data. Also, ask: Tell me about the last time you received EPRP data if not addressed in detail previously.  If this isn’t going anywhere, it might help to focus it a little more:   1. Tell me about the last time you delivered feedback about clinical performance that led someone to change their practice. S-B-O[[2]](#footnote-3): What was the situation? What feedback did you deliver? What was changed? 2. Give me an example of feedback you gave that resulted in little to no change in practice. S-B-O: |
| **Feedback acceptance** | 1. What do you expect people to do with feedback when you give it to them? | Do you (do your clinics, service line, facilities, etc.) develop an action plan for the feedback?  What means do you have for following up to see if any changes have been made? |
|  | 1. What are the consequences of feedback at your facility? (i.e., what happens if someone is given feedback about clinical performance and chooses to ignore it) | What avenues are there if you don’t agree with a piece of feedback you receive? (e.g., appeal, grievance, is it all just handled informally)  How are people held accountable to the feedback they receive? |
| **Feedback seeking** | 1. What could your facility/VISN be doing that they’re not doing now to better track clinical performance? | What could be done to help clinicians better track their clinical performance?  How would those things help you with your clinical performance? |
|  | 1. Is there anything else about feedback that we have not discussed that you would like to share? |  |

1. S-B-O: Situation-Behavior-Outcome [↑](#footnote-ref-2)
2. S-B-O: Situation-Behavior-Outcome [↑](#footnote-ref-3)
